# QTL analyses of drought tolerance in *Eucalyptus* under two contrasting water regimes

**DOI:** 10.1186/1753-6561-5-S7-P40

**Published:** 2011-09-13

**Authors:** Juliana Teixeira, Alexandre Missiaggia, Donizete Dias, Edimar Scarpinati, Juliana Viana, Nadia Paula, Rinaldo Paula, César Bonine

**Affiliations:** 1 Fibria Celulose S.A., Brazil; 2 Faculdade de Tecnologia de Jaboticabal – FATEC-JB, Brazil; 3 Universidade Estadual de São Paulo – UNESP, Brazil

## Background

Drought stress is one of the most important abiotic factors in *Eucalyptus* sp. plantations which influences the growth and limits productivity in cultivated areas, mainly in central, northern and northeastern areas in Brazil, where large parts of these areas have limitations on water supply. The breeders are now looking for tolerant genotypes to overcome this challenge and the QTL mapping approach will help to understand the genetic control of drought tolerance. The objective of this study was to identify genetic loci controlling the phenotypic variation in drought tolerance in a *Eucalytpus* segregant progeny grown under drought and irrigation conditions.

## Material and methods

The progeny used in this study was generated by the breeding program of Fibria Celulose and is composed of 184 F_1_ genotypes from a cross between a tolerant and a susceptible clone to water stress, both *E. grandis* x *E. urophylla* hybrid tree. In a greenhouse condition, the progeny (seedlings with 70 days) was evaluated under two irrigation conditions (1 - control: assigned to a well-watered regime with watering equal to transpiration loss and 2: submitted to water deficit until the onset of initial drought symptoms) in 4 different experiments. Growth (the relative increase in height and stem diameter; leaf number; leaf area; leaf, steam and root dry weight; root-shoot ratio) and physiological traits (net assimilation rate, stomatal conductance, transpiration, instantaneous and intrinsic water-use efficiency, relative water content in leaves, chlorophyll content index, photochemical efficiency and leaf water potential) were measured and BLUPs analysis were performed.Genomic DNA was extracted with CTAB protocol from young leaves and used in PCR amplifications of 121 microsatellite markers. The Onemap software [1] was used in linkage analysis. The estimated BLUPs were used in QTL mapping [2]. The QTLs were mapped by composite interval mapping (CIM).

## Results and discussion

Linkage analysis resulted in 11 linkage groups with 101 markers. The groups were identified using a reference eucalyptus genetic map [3], through common microsatellite loci. The length of the map was 770 cM with an average distance of 7.2 cM between markers. The experimental design allowed analysis of genotype by drought tolerance interaction for the first time in a eucalyptus QTL mapping population, resulting in the identification of 66 loci that control traits under water restriction and 70 loci under irrigation condition, for all of 16 traits evaluated (considering LOD>3.0). Both additive and dominance effects were detected. Around 4 – 7 QTLs were identified for each trait and, in general, the QTLs identified explained from 11% to 30% of phenotypic variation, except by photochemical efficiency, where 59% of phenotypic variation was explained, mainly by 2 QTLs (25.16% and 25.17% and peak LOD score 3.46 and 18.73) mapped in linkage group 6. Most of QTLs identified were specific for each treatment and for just two traits (root and leaf dry weight) QTLs were co-localized for both irrigation conditions and they were mapped in linkage groups 2, 8, 10 and 11. Clusters of QTL for different traits were mapped close to each other at several linkage groups, indicating either a common genetic base or tightly linked QTL. The results are consistent with Ronnberg’s and Yue’s results [4,5],where many QTLs with minor effects are controlling drought tolerance.

## Conclusions

This work revealed the existence of several QTLs that control drought tolerance in Eucalyptus and these QTLs identified may be involved in many tolerance mechanisms that plants can use to avoid this stress. Since clusters of QTL for different traits were identified, potential pleiotropic regulators could be coordinating these traits and these genomic regions can be used to identify key genes for these traits.

**Financial support.**FINEP and Fibria Celulose.
